# 183. Decrease in Invasive Pneumococcal Disease in 7 United States Children’s Hospitals during the COVID-19 Pandemic

**DOI:** 10.1093/ofid/ofab466.183

**Published:** 2021-12-04

**Authors:** Adriana Sarmiento Clemente, Sheldon L Kaplan, William J Barson, Philana L Lin, Jose R Romero, John S Bradley, Tina Q Tan, Pia S Pannaraj, Larry Givner, Kristina G Hulten

**Affiliations:** 1 Baylor College of Medicine, Houston, Texas; 2 Baylor College of Medicine, Houston, TX; 3 Ohio State University College of Medicine and Public Health and Nationwide Children’s Hospital, Columbus, Ohio; 4 UPMC Children’s Hospital of PIttsburgh, Pittsburgh, Pennsylvania; 5 University of Arkansas for Medical Sciences, Little Rock, Arkansas; 6 University of California San Diego, San Diego, California; 7 Feinberg School of Medicine, Northwestern University, Chicago, IL; 8 Children’s Hospital Los Angeles, Los Angeles, California; 9 Wake Forest School of Medicine, Winston Salem, North Carolina

## Abstract

**Background:**

During the 2020 SARS-CoV-2 pandemic, physical distancing and mask use guidelines were implemented resulting in a decline in the number of infections caused by influenza, respiratory syncytial virus and otitis media. A surveillance analysis from England and Taiwan showed a decline in invasive pneumococcal disease (IPD) (Clin Infect Dis. 2021;72: e65-75 and J Infect. 2021;82:296-297). We hypothesized that COVID mitigation efforts resulted in a decrease in incidence of pediatric IPD within the U.S. during 2020 compared to previous years.

**Methods:**

We reviewed all cases of IPD among 7 children’s hospitals from the U.S. Pediatric Multicenter Pneumococcal Surveillance Group from 2017-2020. IPD was defined by the isolation of *Streptococcus pneumoniae* from normally sterile sites (eg. blood, cerebrospinal, pleural, synovial or peritoneal fluid). Pneumococcal pneumonia was defined as an abnormal chest radiograph in the presence of a positive blood, pleural fluid or lung culture. Mastoiditis was identified by positive middle ear, subperiosteal abscess or mastoid bone culture. Serotypes were determined by the capsular swelling method. Hospital admission numbers were obtained for incidence calculations. Statistical analyses were performed using STATA11. A p< 0.05 was considered significant.

**Results:**

A total of 410 IPD cases were identified. The cumulative incidence of IPD (0-22 years of age) decreased from 99.2/100,000 admissions in 2017-2019 to 53.8/100,000 admissions in 2020 (risk ratio 0.54, CI: 0.40-0.72, p< 0.00001). Pneumococcal bacteremia and pneumonia decreased significantly in 2020 (p< 0.05), and although not statistically significant, there were fewer cases of meningitis and mastoiditis when compared to previous years (p=0.08) (Figure 1). Sex, race, age or presence of comorbidities were not significantly different between groups. Most common serotypes in 2020 were 35B, 3 and 15B/C (Figure 2).

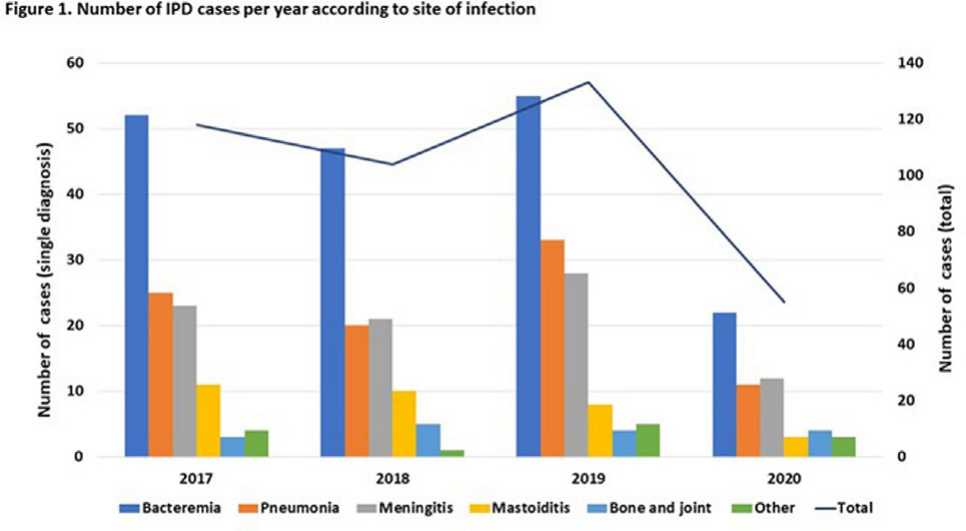

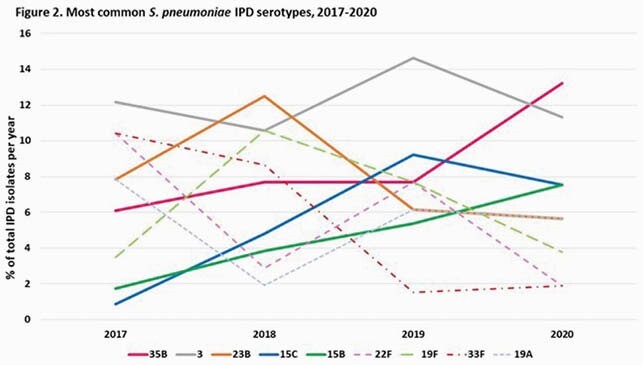

**Conclusion:**

The observed decline in IPD cases during the first year of the SARS-CoV-2 pandemic is likely associated with mask use and physical distancing limiting transmission of *S. pneumoniae* via droplets and viral infections frequently preceding IPD. These precautions might be useful in the future to decrease IPD, especially in high-risk patients.

**Disclosures:**

**Sheldon L. Kaplan, MD**, **Pfizer** (Research Grant or Support) **Tina Q. Tan, MD**, GSK (Individual(s) Involved: Self): Advisor or Review Panel member, Grant/Research Support; ILiAD (Individual(s) Involved: Self): Advisor or Review Panel member; Merck (Individual(s) Involved: Self): Advisor or Review Panel member, Grant/Research Support; Moderna (Individual(s) Involved: Self): Advisor or Review Panel member; Pfizer (Individual(s) Involved: Self): Advisor or Review Panel member **Pia S. Pannaraj, MD, MPH**, **Pfizer** (Grant/Research Support)**Sanofi-Pasteur** (Advisor or Review Panel member)**Seqirus** (Advisor or Review Panel member) **Larry Givner, MD**, **AstraZeneca** (Advisor or Review Panel member) **Kristina G. Hulten, PhD**, **Pfizer** (Research Grant or Support)

